# Book Reviews

**Published:** 1827-06

**Authors:** 


### CRITICAL ANALYSES.

Quæ laudanda forent, et quæ culpanda, vicissim

Illa, prius, cretâ; mox hæc, carbone, *notamus*.—Persius.

*The Life of* Edward Jenner, m.d. ll.d. f.r.s. *Physician Extraordinary to the King, &c. &c. With Illustrations of his Doctrines, and Selections from his Correspondence.* By John Baron, m.d. f.r.s —8vo. pp. xxiv. 624. Colburn, London, 1827.

No eulogy of ours is necessary to exalt the name of Jenner. His philanthropic labours closed one of the "wide yawning gates of death," and his memory will live for ever in the gratitude of the world. If, in days of yore, the civic crown was bestowed upon him who rescued one fellow citizen from destruction, what marks of general adulation would have been deemed sufficient for the universal benefactor to mankind, by whose persevering exertions the desolating career of one of the most dreadful and fatal scourges of humanity was arrested? Honour would have been heaped upon honour, and reward upon reward, and yet the debt of gratitude would have been thought to be poorly paid. Dr. Jenner, it is true, had a sum of money voted for him by parliament; some honours and presents were conferred upon him by foreign countries; and there was no lack of compliment from every quarter of the globe. It is also true that he was looked upon as an honour to the age and country which gave him birth. But still it appears to us, when we reflect upon the mighty benefit he bestowed upon mankind at large, that his rewards were by no means commensurate with his deserts.

Notwithstanding the general attention bestowed upon the important subject of vaccination, and the patient investigations entered into by many able and zealous supporters of the cause, for the purpose of removing every doubt upon the subject, and for the establishment of such rules as might prevent the reputation of vaccination from suffering from any error in the practical application of it, there is yet much discrepancy of opinion upon many important points, which might probably be removed by a more undivided attention to the original doctrines of Dr. Jenner.

In the present work we have not only a sketch of his life,—the history of vaccination is traced from its infancy to its full maturity, and every important fact connected with its origin and subsequent progress is detailed. The work has been composed from materials of the most authentic description; the whole of the notes and correspondence of Dr. Jenner having been given to Dr. BARON by the executors, who were properly of opinion that the task of drawing an accurate delineation of the character and opinions of Jenner could not be confided to better hands. The eulogies of a biographer are sometimes received with doubt, but we know from many sources that Dr. Baron has not been hurried by feelings of private friendship into any commendations which the character of Dr. Jenner did not fully merit. "The world at large has felt and acknowledged the blessings of his great discovery, but few are aware how numerous were his claims to admiration."

Dr. Jenner was nearly fifty years of age before he published his first work on the Variolæ Vaccinæ; and, as the whole of the early part of his life was spent in comparative seclusion, his biographer could not collect materials of much public interest from that period of his existence. His epistolary correspondence with JOHN HUNTER is worthy of attention; for, although Dr. Jenner's replies have been unfortunately destroyed, it tends to show that his opinions and researches were not thought lightly of by that eminent surgeon and physiologist.

After having traced Jenner's history in early life, Dr. Baron has brought together various incidents to illustrate the progress of his mind in effecting the grand discovery of vaccination. From the time of his first successful vaccination in 1796 to the last hour of his existence, he laboured incessantly to disseminate the practice. Although it would have been impossible, and even unjust, to have attempted a sketch of Dr. Jenner's life without dwelling at considerable length upon the important subject which principally occupied his attention, and which has for ever stampt his fame, Dr. Baron wishes it to be distinctly understood that he is not to be considered as the historian of the practice of vaccination.

It is lamentable to know that even Jenner had to contend with a spirit of envy and malevolence, which were calculated to deprive the world of the great advantages of vaccination. We cannot conceal the opinion we entertain upon this distressing part of the subject. It must be granted that much of the virulent opposition by which the beneficent exertions of Dr. Jenner were met in their infancy, arose, not from a laudable conviction that his views or doctrines were erroneous, but from an apprehension that, if he succeeded in bringing his plan to maturity, both his name and reputation would be raised above the common level. Dr. Baron could not pass over some mention of the personal injustice of which Dr. Jenner had to complain in some instances. He has touched upon this delicate subject with moderation, and dismisses it with the following eulogy to the memory of his friend:—"In every private affair, in every public transaction, one principle guided him. The purity of his motives, and the disinterestedness of his actions, have by no means yet been duly acknowledged. Had those who opposed him and vaccination known how little of selfishness, of vanity, or of pride entered into his character, they would, I am persuaded, deeply lament the wounds which they inflicted; and, in the place of bitterness and reproach, would have found cause for unmixed esteem and approbation."

Amongst other reasons adduced by Dr. Baron to show the propriety of publishing this first part of his work without waiting for the completion of the second, it is said that "the recent prevalence of small-pox in different parts of Europe, and the corresponding diminution of confidence in the virtues of the variolæ vaccinæ, rendered it an object of no inconsiderable importance to endeavour to restore and increase that confidence, by showing that Dr. Jenner clearly foresaw the deviations which have been observed; that his doctrines, if properly understood, satisfactorily account for them; and that nothing, in fact, has occurred which does not strengthen and confirm his original opinions, both with regard to the variola and the variolæ vaccinæ."

As might be expected from the remarks made in the preface, the sketch of Dr. Jenner's early life does not occupy much space. It cannot, however, but be interesting to the world to trace every circumstance connected with a man to whom all are so deeply indebted.

"Edward Jenner was born in the vicarage at Berkeley, in Gloucestershire, on the 17th of May, 1749. He was the third son of the Reverend Stephen Jenner, A.M. of the University of Oxford, rector of Rockhampton, and vicar of Berkeley. His mother was the daughter of the Reverend Henry Head, of an ancient and respectable family in Berkshire. This clergyman once held the living of Berkeley, and held at the same time a prebendal stall in the cathedral of Bristol.

Besides his church preferments, the father of Jenner possessed considerable landed property, the family being of great antiquity in Gloucestershire and the neighbouring county of Worcester. It has produced several eminent men; among whom may be mentioned Dr. Thomas Jenner, president of Magdalen College, Oxford, the immediate predecessor of the pious and learned Dr. George Horne. Jenner's father had been tutor to a former Earl of Berkeley; and the late Earl, his brother the Admiral, and indeed the whole of that noble house, always evinced a very strong regard to him and to his family. This excellent and devout man was cut off not long after the birth of his son Edward, at the age of fifty-two, in the year 1754. This heavy loss was, as much as possible, alleviated by the affectionate care and judicious guidance of his eldest brother, the Rev. Stephen Jenner, who brought him up with paternal tenderness. He had another brother, the Rev. Henry Jenner, M.A. Oxon, rector of Rockhampton, Gloucestershire, vicar of Little Bedwin, Wiltshire, and domestic chaplain to the Earl of Aylesbury. From this gentleman are sprung the Rev. George C. Jenner, and Mr. Henry Jenner, who, as will hereafter be seen, assisted their uncle in his interesting pursuits and inquiries.

"Dr. Jenner had three sisters,—Mary, Sarah, and Ann who was married to the Rev. William Davies, rector of Eastington, in the county of Gloucester. He left three sons,—the Rev. William Davies, D.D. rector of Rockhampton; Robert Stephens Davies, Esq. of Stonehouse; and Edward Davies, Esq. of Ebley House, in the same county.

"When about the age of eight years, Jenner was put to school at Wotton-under-Edge, under the Rev. Mr. Clissold. He was next placed under the tuition of the Rev. Dr. Washbourn, at Cirencester, where he made a respectable proficiency in the classics, and laid the foundation of some of those friendships which continued throughout life. His taste for natural history began to show itself at a very early period. Before he was nine years of age, he had made a collection of the nests of the dormouse; and, when at Cirencester, he spent the hours devoted by the other boys to play or recreation in searching for fossils, which abound in the oölitic formation in that neighbourhood. His scholastic education being finished, he was removed to Sodbury, near Bristol, in order to be instructed in the elements of surgery and pharmacy by Mr. Ludlow, an eminent surgeon there. On the expiration of his term with this gentleman, he went to London, to prosecute his professional studies under the direction and instruction of the celebrated John Hunter, in whose family he resided for two years, a favourite pupil." (P. 1—3.)

When Jenner went to London, he was in the twenty-first year of his age, Mr. Hunter in the forty-second. He was not at that time a public lecturer, but he had been about two years a surgeon to St. George's Hospital; and for a considerably longer period he had established his menagerie at Brompton, where he so successfully and perseveringly carried on his inquiries respecting the habits and structure of animals. Jenner was stimulated by the example of such a master to proceed with zeal in various investigations in natural history, to which he was led by all the predilections of his taste, and all the influence of his early habits. It is true that "it was a singular felicity which brought such men together." The pupil not only respected the teacher, but he loved the man.

After completing his professional studies in London, he retired from his preceptor's house; but he did not retire from his good will and affection, nor from his anxious guidance and direction in his scientific pursuits. An uninterrupted epistolary correspondence was kept up between them till within a short time of Mr. Hunter's death. Many of Mr. Hunter's letters are given, and will be perused with interest. They are characteristic of the indefatigable industry of the writer's mind, and at the same time illustrative of the nature and progress of the inquiries of Jenner. Upon these letters Dr. Jenner naturally set a great value.

During the time of his residence with Mr. Hunter in 1771, Captain Cook returned from his first voyage of discovery. The valuable specimens of natural history which had been collected by Sir Joseph Banks were in a great measure arranged and prepared by Jenner, who was recommended by Mr. Hunter for that purpose. He evinced so much dexterity and knowledge in executing this duty, that he was offered the appointment of naturalist in the next expedition, which sailed in 1772. It was his determination, however, to fix his abode in the place of his birth, to which he was partly guided by the deep and grateful affection he felt for his eldest brother, who had been his guide and director when deprived of parental care, and partly by an attachment to the rural scenes and habits of his early life. "Possibly," says Dr. Baron, "in this decision we may now be permitted to trace the agency of a higher power, which induced a young man frequently to reject most flattering prospects of wealth and distinction, that he might be enabled to follow up the leading object of his mind in the seclusion of a country village."

The existence of such an affection as cow-pox was known only in a few districts; it therefore could not become a subject of common observation, nor challenge the keen scrutiny of inquiring intellects to its elucidation.

"Its reported prophylactic powers, it is true, had not altogether escaped popular notice; but no one had arisen to ascertain the correctness of this rumour, or to investigate the source and accuracy of the tradition, till Jenner was led to the pursuit, and, to an almost unlooked-for and unparalleled extent, rendered it available to the subjugation of the greatest scourge of mankind. It is manifest, therefore, that, in the very essence of the inquiry itself, and in the character of the genius of him by whom it was conducted, there was a suitableness and an accommodation, without which it neither could have been begun nor accomplished. This peculiarity will be rendered still more apparent when we come to trace the progress of his mind in maturing the discovery. He mentioned the subject to Mr. Hunter while he was his pupil; and often attempted to arouse the attention of his professional brethren in the country to it, but without success. The merit of persevering in his labours, and the honour of his triumph, rest therefore in an exclusive manner with himself.

"In attempting to unfold character, it is not less instructive than it is interesting to find in the private history of a distinguished individual, the successive links in the chain of events by which it pleaseth Providence to conduct him to that eminence where shines the splendour of his genius and his intellect. This progress in the case of Jenner can luckily be delineated with much accuracy. While yet a youth, and just entering on his elementary studies, that impression was made upon his mind which laid the foundation of all his future researches respecting vaccination; and, with the constancy of a character fitted and fashioned for great achievements, it was never permitted to escape from his consideration till it terminated in that wonderful discovery, the effects of which all nations have enjoyed. It is probable, therefore, that the seed which was sown before his intercourse with Mr. Hunter commenced, would in some future time have germinated, even though he had never witnessed the animating and encouraging example afforded by his prolific and indefatigable genius." (P. 8, 9.)

After his return from London, Jenner commenced the active duties of his profession as a country surgeon. Every leisure moment, however, was dedicated to instructive pursuits. His practice rapidly increased, and he acquired considerable reputation. The study of natural history he still continued ardently to pursue. At this early period of his life he had given indications of genius, which all good judges of character did not fail to recognise as the harbingers of much future reputation. "His knowledge and dexterity as a surgeon, his manners as a gentleman, and his general information as a man of science, rendered his company always acceptable in the families most distinguished by rank or talent in the district where he lived. But there were other qualities, of a personal nature, which more peculiarly endeared him to his intimate associates. He not only commanded confidence by his skill, and respect and esteem by his acquirements, but also secured to himself good-will and affection by the tenderness, and kindness, and benevolence of his nature, and the meekness with which he carried all his faculties in the sight of his fellow men. To much depth and accuracy of observation, and uncommon delicacy of feeling, which at times cast a shade of pensiveness and sorrow over his mind, there was added a liveliness of disposition, which rendered him a friend capable alike of entering into the deepest and saddest emotions of the soul, or participating in all the joys of its gayest and happiest moments." (P. 12.)

As a proof of the amiability and urbanity of Dr. Jenner's manners, and of the powers of his conversation, his friends used frequently to accompany him for miles in his professional rides round the country, and this too even at midnight, that the pleasure derived from his company might be prolonged. He had the happy talent of uniting scientific and original observation with the playfulness, and mirth, and wit of familiar intercourse. His recreations from his more severe studies consisted in the cultivation of polite literature, and occasionally he sought an acquaintance with the Muses. Some specimens of his poetic effusions are given, which, as recreations, are highly creditable to his taste.

We must pass over the letters of Mr. Hunter, which consist principally of questions submitted to Dr. Jenner upon various subjects of natural history and physiology.

Dr. Jenner's interesting and well-known paper on the Cuckoo was read, in March 1788, to the Royal Society, and printed in the Transactions of that year. The most important facts of this communication are detailed by Dr. Baron.

"On the 6th of March, 1788, an event of great moment to him took place. He was on that day united in marriage to Miss Catharine Kingscote, a lady on whom his affections had been long fixed, and in whose counsel and sympathy he found his surest solace in many of the most trying scenes of his future life. She was elegant in her manners, accomplished in her mind, and possessed an understanding of great vigour. She had been an invalid for a considerable time before her marriage, and she never at any time, after her early years, enjoyed robust health. The family of the Kingscotes is one of the most ancient and respectable in this county; and it has received additional distinction from their personal merits, as well as from their alliances with eminent individuals. Anthony Kingscote, Esq. was a kinsman of the great Sir Matthew Hale, and became his guardian after the death of his parents; and the affinity contracted by the union with Jenner will not reflect less lustre on their name." (P. 86.)

His eldest son, Edward, was born on the 24th of January, 1789.

In one of the letters of Dr. Jenner, we find in a few words the burden of the various doctrines so frequently insisted on by writers on Dyspepsia. "You know it has long been my creed that *stomach* is the governor of the whole machine, the mind as well as the body. The seat of action is certainly the brain; but the stomach gives the word of command, and tells it how it shall act."

To Dr. Jenner we are indebted for a knowledge of the laws which regulate the migration of birds.

It is well known that Mr. Hunter died of angina pectoris, and, as a proof of Dr. Jenner's pathological skill, it should be stated that he was the first to suspect, at a very early period of Mr. Hunter's illness, the nature of the malady under which he laboured.

In 1792, Dr. Jenner obtained a degree of doctor of physic from St. Andrews, to relieve himself from the labours of general practice.

In the fourth chapter, Dr. Baron gives the early history of vaccination. Dr. Jenner's attention was drawn forcibly to the nature of cow-pox whilst he was yet a youth. This event was thus brought about:

"He was pursuing his professional education in the house of his master at Sodbury; a young country woman came to seek advice; the subject of small-pox was mentioned in her presence; she immediately observed, "I cannot take that disease, for I have had cow-pox." This incident* rivetted the attention of Jenner. It was the first time that the popular notion, which was not at all uncommon in the district, had been brought home to him with force and influence. Most happily the impression which was then made was never effaced. Young as he was, and insufficiently acquainted with any of the laws of physiology or pathology, he dwelt with deep interest on the communication which had been casually made known to him by a peasant, and partly foresaw the vast consequences which were involved in so remarkable a phenomenon. He was the more stimulated to meditations of this sort by frequent opportunities of witnessing the ravages of small-pox; and by retaining the most vivid and painful recollections of the severe discipline which he himself had not long before passed through, preparatory to his inoculation for that disease. 'There was,' (to use his own words,) 'bleeding till the blood was thin; purging till the body was wasted to a skeleton; and starving on vegetable diet to keep it so.' The possibility of averting such evils could not arise in a mind like Jenner's without possessing it fully, and he resolved to let no opportunity escape of acquiring knowledge on so important a subject. How judiciously, how perseveringly, how successfully, he fulfilled this early resolution, will be seen as we follow him through his various examinations and experiments." (P. 121—3.)

Among other subjects of interest which Jenner carried with him from the country in 1770, when he commenced his studies under Mr. Hunter, was that of cow-pox, which he frequently mentioned. Mr. Hunter never damped the ardour of a pupil by suggesting doubts or difficulties: his answer was, "Don't *think*, but *try*; be patient, be accurate." At first Dr. Jenner tried in vain to rouse his professional friends in the country to push their inquiries upon this interesting subject. It was not till 1780 that he was enabled, after much study and inquiry, to unravel many of the perplexing obscurities and contradictions with which the question was enveloped. In the month of May in that year, he first disclosed his hopes and his fears respecting the great object of his pursuit to his friend Edward Gardner.

* "An incident analogous to that above recorded is mentioned in one of Dr. Jenner's note-books of 1799, in the following words:—

"I know of no direct allusion to the disease in any ancient author, yet the following seems not very distantly to bear upon it. When the Duchess of Cleveland was taunted by her companions, Moll Davis (Lady Mary Davis) and others, that she might soon have to deplore the loss of that beauty which was then her boast, the small-pox at that time raging in London, she made a reply to this effect—that she had no fear about the matter, for she had had a disorder which would prevent her from ever catching the small-pox. This was lately communicated by a gentleman in this country, but unfortunately he could not recollect from what author he gained this intelligence."

"He was riding with Gardner on the road between Gloucester and Bristol, near Newport, when the conversation passed of which I have made mention. He went over the natural history of cow-pox; stated his opinion as to the origin of this affection from the heel of the horse; specified the different sorts of disease which attacked the milkers, when they handled infected cows; dwelt upon that variety which afforded protection against small-pox; and with deep and anxious emotion mentioned his hope of being able to propagate that variety from one human being to another, till he had disseminated the practice all over the globe, to the total extinction of small-pox. The conversation was concluded by Jenner in words to the following effect:—'Gardner, I have entrusted a most important matter to you, which I firmly believe will prove of essential benefit to the human race. I know you, and should not wish what I have stated to be brought into conversation; for, should any thing untoward turn up in my experiments, I should be made, particularly by my medical brethren, the subject of ridicule; for I am the mark they all shoot at.'" (P. 128.)

We must refer to the work itself for the details of the many difficulties Dr. Jenner first experienced, and for an account of the gradual advancement of the grand cause of vaccination. As may be easily imagined, Dr. Jenner's feelings were wrought up to the highest pitch of benevolent gratification when he had fair promise of success.

Dr. Jenner always considered small-pox and cow-pox as modifications of the same distemper, and that, in employing vaccine lymph, we only made use of means to impregnate the constitution with the disease in its mildest, instead of propagating it in its virulent and contagious form, as is done when small-pox is inoculated. Dr. Baron has made some interesting researches upon this question, and we agree with him that they sustain these propositions:

"First, that an eruptive disease, *common* both to man and the inferior animals, has been known in different ages, and in different countries; and that the descriptions given of this eruptive disease by various writers accord so completely with those acknowledged to be characteristic of small-pox, as to render it highly probable that this disease actually existed at a much earlier period than that usually assigned to its origin.

"Secondly, that as there are numberless writers who have described the small-pox in man, so there are others of established name and reputation who have treated of a similar eruptive and pestilential disease, as existing in various countries and in different times among the inferior animals, but especially among cattle; that to this disease they have unhesitatingly applied the name of Variola, and actually recommended such treatment as experience had proved to be useful when that disease attacks man." (P. 163.)

In reference to the returns made by Dr. GREGORY, as physician to the Small-Pox Hospital, which our readers will remember excited much attention, Dr. Baron observes—

"Vaccination seems ever to have been fated to suffer more in character from events within the walls of the Small-Pox Hospital, then from any other quarter: its atmosphere has always been unfriendly to the benign influence of vaccina; but I trust that the inquiry which has taken place will counteract the ill effects that might have arisen, had the statement of the physician remained unexplained. I hope it will not be thought out of place if I express an ardent wish that my professional brethren may be slow to publish fatal or other cases of small-pox after vaccination, until they have good grounds for believing that their patients had regularly and duly passed through the protecting process; and surely there is no reason to think that this had taken place in any of the fatal instances reported by Dr. Gregory, as a reference to his examination will fully evince." (P. 274.)

We do not think the censure upon Dr. Gregory merited; for, whatever may have been the case with some other physicians to the Small-Pox Hospital, the whole tenor of *his* writings has been in favour of vaccination, and we know that practically it has in him a most zealous promoter. The cases alluded to were instances of small-pox after imperfect vaccination.

The disgraceful attempts which were made by certain individuals to rob Dr. Jenner of the merit of the discovery of vaccination, have been frequently and properly commented upon. Dr. Baron treats this part of the subject with much temper and good sense. As the biographer of Dr. Jenner, it was his duty, however, to state without reserve every fact connected with the history of vaccination. And there can be no doubt that there were many persons whose whole endeavours were exerted to sink the name of Jenner as much as possible, and to attach to themselves the reputation for a discovery to which they had not the shadow of a claim.

It may be of importance to remember that Dr. Jenner invariably maintained that any cutaneous disease, however trifling or slight in appearance, was capable of interfering with the regular course of cow-pox, and of preventing it from exercising its full protecting influence. We have had many opportunities of verifying this observation in our own practice.

At a subsequent period, Dr. Jenner was particularly anxious to send out supplies of virus to our distant possessions in the East. Government were not very cordial in their assistance, and to the honour of Dr. Jenner let it be mentioned, that he desired his own name to be put down for a subscription of one thousand guineas. Vaccine matter, however, was forwarded from Vienna to Constantinople, and from thence to Bombay; and his philanthropic design was thus rendered unnecessary.

As some proof of the principles upon which vaccination met with support and countenance in some places, Dr. Baron relates the following anecdote:—"I believe it was at this time that the incident I am about to mention occurred. Notwithstanding his repeated notices of gratuitous aid, one parish had hitherto obstinately held back. This year, however, he found the people bringing their children in great numbers. Of course he wished to know by what means they had become converts to the new inoculation. He found that arguments of a very authoritative nature had brought about the change. The small-pox, in the course of the preceding year, had been introduced into the parish, and proved extremely fatal; but it was not this circumstance, nor yet the security of those who had been vaccinated in the adjoining parishes, that brought cow-pox inoculation into favour. The cost of coffins for those who were cut off by small-pox proved burdensome to the parish; the churchwardens, therefore, moved by this argument, effectually exerted their authority, and compelled the people to avail themselves of Dr. Jenner's kind offer!" (P. 433.)

The almost undivided attention which Dr. Jenner paid to his favourite subject necessarily diminished his practice as a physician, and it was thought that the magnitude of his discovery, and the very disinterested manner in which he had sacrificed his time and property for the general good, were fit subjects for the consideration of parliament. Preparatory to a direct application to the great national council, some of the chief personages in Gloucestershire began to take measures to give some testimony of the value in which Dr. Jenner was held by those who knew him best, and amongst whom his life had been spent. The late Earl of Berkeley took the lead in this becoming expression of public feeling; the Countess of Berkeley also applied herself, with her usual earnestness, to effect the object.

In the parliamentary investigation which took place for the purpose of considering the claim of Dr. Jenner to national reward, we again find attempts were made to diminish his reputation, and to defeat the object of his friends.

"The documents presented by Dr. Pearson were evidently intended to prove that vaccine inoculation had been practised by others before Dr. Jenner. His second examination before the committee had a different object. It went to show that, though Dr. Jenner promulgated the practice of vaccination, he really knew very little about the matter; that his opinions as to its origin were erroneous; and that it required the experiments and labours of other observers to correct his mistakes; and that he and Dr. Woodville had the chief merit in the establishment of the vaccine inoculation. Thus, after making due allowance for the claims of Farmer Jesty, and the valuable and scientific investigations of others, nothing was left to Jenner save that of being the publisher of a provincial rumour, the nature of which he himself did not fully understand! What must have been the feelings of those who could utter statements capable of leading to such inferences?" (P. 501.)

After much discussion, the sum of 10,000*l.* was voted to Dr. Jenner. "Thus we have seen," says Dr. Baron, "in what manner the assembled representatives of our country, supported by the declaration of the King's minister in his place in parliament, rewarded the discovery of Jenner."

In the course of the parliamentary investigations, many of the most distinguished members of the profession very highly eulogised the liberal conduct of Dr. Jenner.

We believe there was but one opinion respecting the amount of the grant made. It was almost universally deemed inadequate to his powerful claims to public gratitude. In the early part of the preceding century, the House of Commons had voted a much larger sum for importing a silk-throwsting machine from Italy.

We pass over with due contempt the ridiculous and insignificant efforts of M. Husson to show that his countryman, M. Rabaut, had a prior claim to the discovery of vaccination.

The fourteenth chapter gives a history of the formation of the Royal Jennerian Society. Here again intrigue was busily employed to place others in the distinguished post of head of the society, to which honour Jenner alone could have a claim. These unworthy efforts, however, were completely defeated.

The fame of Dr. Jenner must always maintain the highest rank, not only on account of the splendid discovery he made, but from the very liberal and disinterested manner in which he at once stated his views to the profession and public. This fact is alone an answer to the various insinuations that have been sometimes hazarded to lead to the inference that personal interest was his chief object. It was properly and fairly urged, during the parliamentary investigation, by Sir Walter Farquhar, Mr. Cline, and other leading members of the profession, that Dr. Jenner might have made at least

10,000*l.* a-year, had he kept within his own breast the preservative powers of vaccination.

In confirmation of the eulogies Dr. Baron has so warmly bestowed upon the pure philanthropy of Jenner's mind, we may state that we have been assured by a distinguished member of the Berkeley family, with whose countenance, patronage, and friendship Dr. Jenner was honoured for many years, that nothing could exceed his anxiety when his experience of vaccination had not led to any positive and satisfactory conclusions, lest the promulgation of the practice should prove injurious, by inducing parents to forego the advantages of small-pox inoculation, and thus expose their children to the natural disease. He was frequently in the habit of exclaiming, that "he should either prove a blessing or a curse to his country!"—a blessing, if he succeeded in the hopes he so fondly cherished; but a curse, if, from mistaken zeal, he should be the means of proposing vaccination as a preservative, and subsequent experience should prove that it afforded no protection against the ravages of small-pox.

The manner in which Dr. Baron has performed the duty he has undertaken, is highly creditable to his ability and judgment. But, notwithstanding the reasons assigned at the beginning of the volume, we are of opinion it would have been more judicious to have published the whole of the work at once, even if some delay had been unavoidable. Its success would certainly have been greater, and the interest in the perusal of it uninterrupted by a break which the title-page does not lead the purchaser to expect.

---

*A Critical Analysis of the Memoir read by* Dr. Barry *before the Academy of Sciences, on the 8th of June,* 1825, *at the Institute of France, on Atmospheric Pressure being the principal Cause of the Progression of the Blood in the Veins.* By Henry Searle, Surgeon.—8vo. pp. 88. Callow and Wilson, London, 1827.

Dr. Barry's researches on the influence of atmospheric pressure on the circulation have lately excited considerable attention, and, as might be expected from the nature of the subject, great contrariety of opinion. Having in a former Number given a sketch of the opinions of Dr. Barry, it is incumbent upon us to notice the critical examination of them by Mr. Searle.

In all philosophical inquiries, the utmost coolness of discussion and temperance of expression should undoubtedly be preserved, and, without accusing Mr. Searle of a direct violation of either the one or the other, we certainly discover in his preface a little of that critical tartness which, if replied to in the same tone, might very probably ruffle the even spirit with which inquiries of this sort ought to be conducted. Mr. Searle informs us that "not one of Dr. Barry's experiments, by which he has made so many proselytes to his theory, will bear the least examination: they are merely proofs that there has been unnecessary pain inflicted." Now, as there are doubtless "many proselytes" to the theory of Dr. Barry, who have attentively investigated his experiments, and the results deducible from them, it is going too far to say "that they will not bear the least examination." They may be, and we think are, fallacious; but yet they are sufficiently plausible to have caught the approbation of some of the most distinguished physiologists in France, and to have excited very general attention in this country.

Dr. Barry has made considerable inquiry whether his theory has ever occurred to the minds of any other persons? and Mr. Searle replies, "I can in veracity assert that it did present itself to me about four years ago, and most probably it has to others: but he was not likely to find in print any thing so palpably discordant with some of the plainest phenomena in the animated part of the creation."

Mr. Searle observes also, that Dr. Barry has openly said, in the presence of a number of persons, and within his hearing, at the Hunterian Society, that it is not fair to judge of his theory by his book; and that, if we would suspend our objections until the appearance of his second Memoir in the spring, that we should be perfectly satisfied of its truth. Now, if this be the fact, (and we have no reason to doubt it,) it would obviously have been better that Mr. Searle should have delayed his critical examination until the theory was presented to us in a more perfect form. We are obliged to Mr. Searle for the mention of this fact, as we certainly should not feel ourselves justified in formally analysing or criticising the presumed refutation of opinions, which the author himself confesses to require still further development before we can judge of their validity. We have merely to state, then, for the information of those who may be interested in the subject, that the experiments of Dr. Barry are examined by Mr. Searle with much attention, and that the results which have been drawn from them are deemed erroneous.

In reference to the fourth experiment of Dr. Barry, which is quoted at length, Mr. Searle makes the following observation: "The laboured anatomical inquiry apparent throughout this quotation commands a degree of respect: at the same time it must be regretted that so much pains should have been taken in the support of error, rather than in the development of truth; for it is palpably evident that Dr. Barry has not read impartially the anatomical language of nature, but has had a particular object to accomplish, and has made a stretch of contrivance in taking this peculiar view of the mechanism of the pectoral viscera, in violation of the real laws under which it is governed."

We confess that the following experiment made by Mr. Searle offer objections to the doctrines of Dr. Barry, which we have not ingenuity enough to remove.

"But, in order to obtain an unequivocal proof that the circulation of the blood is perfectly independent of the aid of a vacuum forming around the heart, an experiment was made, which probably could not have been more simple and apparent, nor less imitative. I removed sufficient of the parietes of the thorax over the region of the heart of a fully grown rabbit, to enable me to introduce a finger, and raise the heart upon its base, and give any direction to its long axis. Here was an influx of atmospheric pressure directly around this organ, where the vacuum ought to occur; the impossibility of its occurrence need not be urged. This animal bled profusely from the wounded extremities of two or three intercostal arteries, which exhausted it for a time; it afterwards ate, ran about, and lived seven hours; when it was killed, to prevent any suffering taking place from the inflammation which would have supervened." (P. 37.)

We have been informed also, by one of the most distinguished experimental physiologists of the day, that, if the ribs and sternum are entirely removed, the circulation still continues for a time, in an equable and natural manner. We know also that Dr. Barry denies this fact; and, as we have not ourselves made the experiment, it is impossible for us to determine which party has been led into error by experiments imperfectly conducted, or deductions hastily and incorrectly drawn.

At the conclusion of his analysis, Mr. Searle observes that "It cannot be disputed that Dr. Barry has invited the attention of the faculty to a mode of treating poisoned wounds, which had perhaps entirely fallen into oblivion. Although this be not a discovery, it is tantamount to one. Its practical utility cannot at present be estimated, nor will it be duly appreciated until his theory on the circulation of the venous blood has become silenced; for it has been so strenuously forced upon the minds of the faculty, that its assumed importance eclipses, in a measure, the great good which Dr. Barry is likely to occasion by his experiments on poisoned wounds. It must be admitted that Mr. Ellerby has made this practice more extensively useful, by having ascertained that pressure in any form, applied near the part, so as to intercept the wound and the heart, will answer the same end as the cupping glass; for there is no inhabited part of the globe where a glass, a cup, or some kind of vessel, might not be promptly found, for the purpose of preventing any absorption of the deleterious matter, until the excision or destruction of the part could be made.

"In conclusion I must be allowed to say, that the replies I have made to some of Dr. Barry's remarks in his preface, and the views taken of his experiments, are such as, in my humble opinion, are directly called for, and should not be passed by, for obvious reasons: at the same time, I beg to express a very high sense of Dr. Barry's merits, in having so ably and decidedly placed before the public the practice which ought to be adopted in all cases of poison; and, if to save lives is an important improvement in medicine, Dr. Barry has a right to claim an universal observance of this well-known motto—*Palmam qui meruit, ferat.*" (P. 87.)

Since the above was written, we have met with the following passage in a very interesting work on Physics, lately published by Dr. Arnott, and which we think too much to the point to require any apology for its insertion.

"Some recent authors, as stated above, either not being aware of the facts which prove that the blood is every where pressed into the veins from the capillaries, with force much more than sufficient to raise it to the heart again; or being unable, from their little familiarity with physics, to draw exact conclusions from the facts, or to avoid errors in their own hypotheses, have promulgated the opinion that the progression of the blood in the veins is greatly owing to a suction power in the heart or chest: that is to say, to the atmospheric pressure remaining constant on the body generally, while it is occasionally lessened about the heart; a circumstance, of which the whole effect, as stated above, is merely a slight disturbance of the uniformity of the venous current near the chest. Now such a doctrine could not be proposed or entertained for a moment by a person understanding the principle of a common household pump; and that it has been published and tolerated by able men in the present time, will remain a proof to posterity of the deficiency, as regards fundamental science or natural philosophy, which now exists in the ordinary medical education. Much ingenuity has been wasted upon it, particularly by Drs. Carson and Barry; the latter of whom, after laborious investigations, by experiment on living animals, has even attempted to build upon it a superstructure of medical theory and practice. The fault, however, may be less in the parties who have been pursuing what appeared to them an important object, than in the system of education which left them exposed to such errors. Dr. Barry need not blush to have proposed an explanation, in which members of the French Institute, sitting to judge it, were not prepared clearly to point out the fallacy. To say that the influence of the heart or chest is the power which draws the blood to the heart from the general system, is just as if one asserted that the ocean tide at the mouth of a river is the power which collects the tributary streams in the interior country.

"We shall enter into a little detail on this subject, because the discussion will elucidate some minor points connected with the circulation.

"Presuming, then, that the reader perfectly understands the theory of pumps, and therefore of atmospheric pressure, as explained under Pneumatics, he will readily understand the two following propositions, either of which proves it to be a physical impossibility that a sucking action of the heart or chest can be a cause of the blood's motion along the veins. 1st. The veins are pliant tubes free to collapse, and no pump can lift liquid through such. 2d. The *suction power* of the chest in ordinary respiration is too weak to lift liquid a distance of even one inch through tubes of any kind.

"A practical illustration of the first proposition is afforded by putting the point of a syringe, capable of making a complete vacuum, if desired, into a piece of gut, or eel-skin, or vein, filled with water, and then trying to pump up the water. The result will be, that the fluid close to the mouth of the syringe will enter it, and the sides of the pliant tube will then collapse as a valve against the syringe, making an end of the experiment. In exact proportion to the rigidity of the tube would be the distance to which the influence of the syringe would extend in it: if it required, for instance, half an ounce of pressure on the square inch of its surface to make it collapse, then the pump would draw up one inch of water, and so for other proportions. If, during the action of the syringe, the tube were allowed to open at the bottom into a vessel of water, instead of the syringe then drawing any more water from the vessel into the tube, the original contents of the tube would straightway be discharged downwards into the vessel: and the result would be the same even if there were a thousand tributary streams pouring into the tube, unless they entered with force enough to rise up to the syringe.

"The explanation of all these facts is found in the pressure of the atmosphere, seeking entrance every where at the surface of the earth, with a force of fifteen pounds per square inch, and overcoming any opposing force less than this: sufficient, therefore, to push a column of water thirty-four feet high through a rigid tube into the vacuum of a pump, but causing the sides of the tube to collapse, unless able to sustain at any given part a compressing force equal to the weight of water in the tube below.

"Some bad reasoners on this subject have believed that if a suction power exist equal to one inch column of liquid, any column, however long, must follow the first inch when acted on by the power in question; for, say they, the atmospheric pressure preventing a vacuum will prevent separation of the liquid. Now this reasoning is altogether inapplicable to pliant tubes, because the ready collapse of their sides will both allow the separation and prevent the vacuum; and, with respect to rigid tubes, it is equivalent to asserting that a force just capable of lifting one link of a chain, must therefore be able to lift any number of connected links. Water in a rigid tube, to which air has no admittance, may be considered as a chain, for it is held together by a force of fifteen pounds per inch pressing inwards at the two ends: and any force less than this cannot therefore lift one portion of it away from another, and therefore cannot draw out a drop but by lifting the whole. A man cannot suck water from a full rigid tube which is closed at the bottom; and if the bottom be open, and he has not power to support the whole contained fluid, it will sink from its tantalised lips, to stand at an elevation marking his suction power.

"To illustrate the second proposition respecting the trifling suction power really residing in the chest, we shall state that a person of ordinary strength, using the power of the chest only, and not of the mouth separately, (which is a smaller and much more powerful pump than the chest,) cannot through a rigid tube suck water from more than about two feet below: and the opposite action of blowing outwards has nearly the same limit as is found by dipping the end of a tube two feet into water, and then trying to blow through it. Now, as water rises more than thirty feet towards a perfect vacuum, such as that of a good pump, the facts mentioned prove that the diminished pressure in the chest, as an approach to a vacuum, is never more than *one-fifteenth* of the whole, and the increased pressure during straining is in a corresponding degree. But in ordinary breathing, instead of differences corresponding to a liquid column of two feet or a *fifteenth*, the increase and diminution of air-density in the chest is measured by a column of less than one inch, or about a *five-hundredth*. This is easily shown on breathing through the nose, and holding one end of a glass tube in the mouth while the other end is immersed in water, by noting how much the water in the tube rises above the surrounding level during *inspiration*, and sinks below it during *expiration*. The mouth during this experiment may be considered as part of the general cavity of the chest, to and from which air is passing by the narrow openings of the nostrils. In tranquil breathing, with both nostrils open, the fluctuation in the tube is less than half an inch each way; with one nostril closed, and the other a little compressed, it may amount to a whole inch; and with hurried or convulsive breathing, like that of an animal in terror or in pain, it may exceed twelve inches. Although the measures so obtained from the mouth are somewhat too small for the changes in the chest itself, because the chest is more remote from the opening by which the external air enters, the difference is very trifling, as is proved during such experiments by stopping the nostrils altogether, and continuing the same respiratory efforts; and also by the agreement of the results with strict calculation founded on the inertia and velocity of the air respired: a calculation similar to that required in adjusting the index to the machine for measuring water-currents. In common healthy breathing with the mouth open, the fluctuation of pressure in the chest is measured by less than half an inch motion each way of the liquid column. Dr. Barry, not aware that this point could be so easily determined by the bloodless experiment described by the author above, or even by a simple calculation, has sought the solution by numerous trials on living animals, into some part of whose chest he forced a tube: but, even if farther experiments had been at all necessary, these of Dr. B. could not have decided the question. 1st. Because the breathing became violent or unnatural, from the pain and agitation of the animals; and, 2d, because the experimental tube often or always became a syphon: and Dr. B. not adverting to this fact, has not recorded the difference of level in the liquid at the two ends. That the external level was for the most part higher than the internal, is proved by his having noticed almost solely the *inhaling* action of the chest, although the *exhaling* is generally an equal, and often a more powerful effort.

"Calling an inch column of blood, then, the measure of the greatest sugescent and repellent powers of the chest during ordinary respiration, we see that the force which really sends the blood from below to the heart may have to lift a column one inch shorter during inspiration, and one inch longer during expiration. And this is the full and true measure and nature of the influence of the respiration on the blood's return to the heart by the veins. To say that the atmospheric pressure, modified by respiration, is the great power which moves the venous blood, is as if we said that a boy, standing near the ponderous fly-wheel of a steam-engine, and giving it his Lilliputian thrust alternately backward and forward, were the prime moving force of the machinery.

"The truth explained above, that no kind of pump can lift fluid through pliant tubes, free to collapse, like the veins, renders it unnecessary to speak farther here of the pumping action of the heart, insisted on by Dr. Carson, or of that other action to which also he attributes great influence,—viz. the tendency towards a vacuum external to the lungs and around the heart, produced by the disposition of the lungs to collapse. It may be remarked, however, that this last influence is more considerable than the simple inspiratory action dwelt on by Dr. Barry, and operates during expiration nearly as much as during inspiration, varying in force with the degrees of expansion of the chest. It is weaker in the living than in the dead body, because the rigidity of the distended pulmonary arteries helps to support the weight of the lungs.

"Were it necessary to give proofs to persons unable to follow the above argument, that a suction power in the heart or chest is not the force which draws the blood from the extreme veins, the reference is ready to many notorious facts quite incompatible with that supposition; for instance—A vein tied, fills tensely below the ligature; a vein cut across bleeds from its distant orifice, and will fill a lofty tube connected with it; the circulation is perfect in the fœtus in utero, which breathes not; and it goes on in persons holding their breath, and in divers, &c. &c.

"After the explanations now given, it is almost superfluous to remark that *absorption* in animals cannot depend on atmospheric pressure, and that the effect of cupping glasses employed to extract blood, or to prevent the absorption of poison in wounds, in no way depends upon the fluctuating density of the air in the chest. Dr. Barry's reasonings upon these subjects involve the same fallacy as his reasonings on the venous current."

---

**Observations on the Treatment of Gonorrhœa by a new Preparation from the Balsam of Copaiba; with illustrative Cases.* By James Thorn, Member of the Royal College of Surgeons.—Highley, London, 1827.

Mr. Thorn has devoted considerable attention to the effects of Copaiba in Gonorrhœa, by which he has been led to the discovery of a new preparation of that substance, which he strongly recommends. Never having tried the "extract," we cannot give any opinion from our own experience, and shall therefore content ourselves with making our readers acquainted with the author's statements. Experiment alone can decide the question.

Having spoken of the inconveniences attending the Balsam of Copaiba, as usually given, the author continues:

"From these circumstances, I was induced to try what would be the result of an analysis of the balsam, conceiving that its active principle might be very much concentrated, and rendered more extensively useful when separated, than in the present form. By distillation, an essential oil was produced, of a light green colour, having a most unpleasant smell and taste; its specific gravity being 0876 to 1000 water, leaving a brown resinous extract, quite soft, but becoming hard and brittle when cold; nearly tasteless and inodorous, soluble in æther and pure alcohol. The proportions obtained from two ounces of the balsam of copaiba were—eleven parts essential oil to five of extract, the respective merits of which I shall hereafter mention; and from the result I feel convinced that, by separating the essential oil from the resinous extract, a very irritating and obnoxious part of the balsam is got rid of; and, moreover, in the extractive resin all the virtues of the copaiba reside.

* This and the succeeding article were intended for the Collectanea, but, having been set in the wrong type, we have inserted them here for the sake of uniformity.

I am not aware of any mention having been made by writers on Materia Medica, as to the medical qualities of either the essential oil or the extract from the copaiba. In THOMSON's Dispensatory, page 295, fourth edition, in speaking of balsam of copaiba, there is the following observation: "By destructive distillation, it (the balsam of copaiba) yields some empyreumatic brownish-red oil, an acidulous water, carbonic acid gas and olefiant gas, but does not yield benzoic acid:" though, in his Conspectus, page 49; under the article of Balsam of Copaiba, the composition is mentioned as resin and volatile oil. The method by which the oil has been separated from the resin has been by dry or destructive distillation, and the oil thus produced was of a light green colour: but it was found necessary to be very careful as to the application of heat; and I can well conceive the colour of the oil, as stated by Dr. Thomson, to have arisen from the excessive heat employed in destructive distillation causing the extractive resin to come over with the essential oil, and thereby imparting to it the brownish red colour described.

In the preceding remarks on the new preparation from the balsam of copaiba, and in giving the cases in which I have tried its efficacy in gonorrhœa, and its superiority over the common form, I have merely stated facts as they have come under my notice within a very short period of time. There are some few advantages, however, as a therapeutic agent, the extract possesses over the balsam, which it may not be amiss to mention more fully than has been done in any former part of this work. The first, which is that of its not producing the nausea, any disturbance of the alimentary canal, nor the eruptions on the skin, which the balsam does. Indeed, in no instance in which I have given the extract have I seen any uneasiness produced by its exhibition: the superiority on this account was fully proved in the case of a gentleman, who requested me to prescribe for him on account of a gonorrhœa from which he was suffering; but coupled his request with the remark, that, having so frequently tried to take the balsam of copaiba in every variety of form that could be thought of, which his stomach had refused, he would rather continue to suffer from gonorrhœa than again attempt taking it; and, to use his own words, "it played the very devil with him." I determined, however, on trying what would be the effect of the extract of copaiba on this gentleman's irritable stomach. I accordingly gave him ten grains three times a-day, and the result was quite as satisfactory as in any of the preceding cases which I have cited, without producing the least sickness or any uneasiness of the stomach. The form of pills, and its being nearly tasteless, are also great advantages attached to the extract, from the facility that is afforded in increasing the dose of the medicine to a considerable quantity with so little inconvenience to the patient. In forming a comparison between the treatment proposed in the preceding observations and the usual modes of managing gonorrhœa, there must appear a decided improvement in a plan by which the patient is relieved with so little personal inconvenience. It may, however, be supposed that, in recommending the extract of copaiba in the different stages of gonorrhœa, I am holding out to the medical public a wonder working drug, which will supersede all those regulations which are so necessary when any of the secretions are inordinately affected. It must be obvious to every one acquainted with the phenomena of disease, that each affection has not a substantive existence of itself, and that the failure of cure depends on the appropriate remedy not being discovered: but that there are certain conditions on which each form of disease depends, and that a minute attention to diet, temperature, exercise, and with the use of such medicinal agents as have a known effect, can alone give the probability of success of cure. In the treatment of gonorrhœa, in whatever stage it may be, I invariably caution the patient against the use of stimulating drink: of so great importance is it, that the extract of copaiba will appear a perfectly inert preparation, unless the patient is restricted in this particular.

Mr. Tyrrell has done me the favour of trying the effects of the extract of copaiba, and given the results, and his opinion of the preparation, in the following letter:—

"According to your wish, I send you the result of those cases of gonorrhœa in which I have had an opportunity of trying your new preparation of copaiba; and I take the liberty of adding a few observations on the disease, and the ordinary modes of treatment. I shall not enter into a minute detail of each case, but merely give those points which appear to me most worthy of notice, and which I hope will be sufficient for you.

"1st Case, ætatis nineteen; first gonorrhœa. Discharge moderate and greenish; slight ardor urinæ; chordee had existed three days. To take Pil. Cal. c. Colocynth, grs. x. statim; to abstain from malt liquor or spirits. Ext. Copaibæ grs. x. (in pil.) ter quotidie.—Cured in three days.

"2d Case, ætatis twenty-one; second gonorrhœa. Discharge profuse; ardor urinæ, and occasional chordee, existed six days. To take Pil. Calom. c. Colocynth. grs. x. statim; to abstain as the former. Pil. Extr. Copaibæ grs. x. ter quotidie, increased to grs. xv. on the fifth day.—Cured in seven days.

"3d Case, ætatis twenty; first gonorrhœa. This young man had been for some days under my care for gonorrhœa, which had existed for about three weeks before he applied to me: he had been taking the Cubebs without relief. I gave him the Balsam Copaiba with Sodæ Subcarb. and mucilage, but was obliged to omit it, as it acted so much on his bowels. His discharge was profuse, with slight ardor urinæ, but no chordee. To take Pil. Ext. Copaibæ grs. x. ter quotidie; to abstain as the other patients.—Cured in six days after commencing the pills.

"4th Case, ætatis forty; first gonorrhœa. Discharge but little, no ardor or chordee; discharge greenish, has existed two days. To take Pil. Cal. c. Col. grs. x. statim; Pil. Extr. Copaibæ grs. x. ter die; to take one glass of wine, having been used to six or eight daily. Has been five days under treatment; discharge only apparent in the morning, but not quite well.

"5th Case, ætatis fifteen. This boy had suffered from gonorrhæa for three months: he came to me with gonorrhœa, phymosis, and inflamed prepuce. Ordered

Catapl. Lini frigid. c. Lot. Saturni; Pil. Cal. c. Colocynth. grs. x. altern. noct.; to keep recumbent, support the penis and scrotum; to inject the lotion under the foreskin. In a few days the inflammation and phymosis subsided, leaving a profuse gonorrhœa, without ardor or chordee. To take Pil. Extr. Copaibæ grs. x. ter die.—Cured in five days.

"These are the only cases of which I can give a fair report. Two others have taken the pills: one I have entirely lost sight of, and the other has been to me only once (two days ago.) You will observe that, in the cases of short duration, I have been careful to act on the bowels before giving the extract of copaiba, and also that I have restricted the diet in part. I have, in fact, adopted the same plan as when prescribing the balsam itself; and in very many cases I have found the balsam equally efficacious, but there are many objections to its employment which appear to me not applicable to your preparation. Not any of the cases related here have been acute, so that I cannot speak of the utility of the extract in such cases; but my own experience in the treatment of gonorrhœa would not lead me to prescribe it during the existence of the high inflammatory stage of the disease."

---

### Mr. Fay *on an Improved Forceps for the use of Dentists and others.*—1827.

Mr. Fay, a dentist of some repute, has recently adopted a method of cutting the teeth across when they are decayed, instead of extracting them. Without offering any opinion, we subjoin his statements as given in the Transactions of the Society of Arts.

Having early in my professional career felt the greatest inconvenience from the imperfections of the instruments usually employed for the extraction of teeth, I was induced to prepare others more accurately adapted to their figure. From an attentive consideration of their structure, I was soon convinced that the *neck of the tooth* was the only part on which the necessary force could be applied with the greatest safety and advantage. Now, as the teeth do not all present the same configuration, it follows that the figure of their necks must also vary; of this fact every anatomist must be convinced. I state nothing new when I say that there are certain teeth formed very much alike, both in the upper and lower jaw, and that of the teeth thus similar to each other there are various classes, but that each class retains the same peculiarity of figure in all ages, and under all circumstances. Having stated thus much, which I think necessary in order to prepare you for what follows, I beg leave to say that I have invented a set of instruments accurately, because anatomically, suited to these several classes of teeth, a desideratum, as I believe, never before accomplished. These instruments are forceps, corresponding in number to the different classes of teeth, and suited to the same classes on the right side, and on the left, in the upper and in the lower jaw, amounting to six in number; but, as it is necessary to have two sizes of three of these, the number of extracting forceps is nine, and, by referring to the plate, it will be seen with what precision the forceps may be applied to the proper tooth. Thus I found myself in possession of a set of instruments answering the required end, because correctly adapted to the varied form of the parts to which they are intended to be applied.

The next point was to determine how the power thus possessed should be directed. The line of least resistance is that which coincides the nearest with the average direction of the axes of the roots: consequently the power should be applied more or less perpendicularly to the jaw-bone.

I do not pretend to say that I am the first person to attempt the perpendicular extraction of teeth; but I hope to show to the Society that I have accomplished that object with the simplest, the safest, and the easiest means.

The advantages which I consider these forceps to possess over all others, are briefly these:

1st. They may, as before stated, be accurately applied to the necks of the several classes of teeth: they are made to fit the necks only, never making the least pressure on the enamel or body of the teeth, and consequently may be used without any danger of breaking a carious tooth in the attempt to extract it.

2d. They never can slip when once accurately applied on the necks of the teeth; a great practical benefit.

3d. No cutting of the gum, or any other preparatory measure, is necessary, as the edges of the blades of the forceps may be at once brought upon the necks of the teeth.

4th. A provision is made by the beaked form of the extremities of the blades of the forceps designed for the extraction of the teeth having more than one root, by which means the forceps may be steadily fixed on the remains of a decayed tooth, even when the edges of such teeth are below the level of the gums. To which may be added, that they enable the operator to extract the teeth in the perpendicular direction with a less amount of force than any other instruments.

The manner of using the right-angled forceps is shortly this:—The forceps having been carefully applied on the neck of the tooth to be removed, the operator is to put a small bit of any light wood across the jaw, of a thickness sufficient to occupy the space between the joint of the forceps and the anterior teeth; then steadily seizing the handles of the instrument, he is to make a gentle semi-rotatory motion at the same time that he is pressing the handles of the instrument downwards. This motion separates more easily the vessels and membranes connecting the roots to their sockets, and surrounding the neck of the tooth; for, be it remembered, the soft parts, and not the hard, present the greatest resistance to the removal of the teeth. It might be supposed that the pressure of the piece of wood on the anterior teeth, in loosening the diseased one, would produce pain: on the contrary, it is not felt, because the action and reaction are exactly equal between the pressure on the jaw and the resistance of the tooth.

---

*An Introductory Lecture on Human and Comparative Physiology. Delivered at the New Medical School in Aldersgate-street.* By PETER M. ROGET, M.D. F.R.S. &c. Consulting Physician to Queen Charlotte's Lying-in Hospital; and senior Physician to the Northern Dispensary.—8vo. pp. 103. Longman and Co. London, 1826.

WE have always been of opinion that sufficient attention is not devoted to physiology in the course of medical education generally adopted in this metropolis, and we therefore feel pleasure in directing the notice of our readers to any thing calculated to remedy this evil. We have, indeed, made it our business to lay before the profession some account of most physiological works which have appeared within the last few years, within as short a term as possible after the date of their publication; and we are induced to notice the little volume before us because, as it was originally intended as introductory to a course of Lectures expressly on this subject, so it may, with equal propriety, be looked upon as a general introduction to any of the more extensive works, of which we have given analyses in previous Numbers. It is, in fact, an exposition of the objects and scope of Human and Comparative Physiology,—of their connexions with other sciences,—of their high rank, and unquestionable utility.

Anatomy is the first step towards all medical knowledge; it is the groundwork on which the whole superstructure is to be raised. This is universally admitted; but still it is only the groundwork, and is of little value unless the superstructure *be* raised; and we suspect that too often the student aims only at possessing this foundation, and leaves those sciences of which it forms the base for future acquirement. Dissection only shows that the body consists of various parts, "some hard, some soft, and others fluid;" but, viewed without relation to function, the most intimate acquaintance with structure is but barren and useless knowledge.

"Thus, while we confine our attention to the mere anatomy, all is perplexity and disorder; we are overwhelmed by the multiplicity of objects, and lost amidst the mass of unconnected details. But no sooner do we study the parts of the animal frame with reference to their uses, and their subserviency to the functions of the living body, than the whole appears under a new aspect. New light is thrown upon every branch of the subject, and new interest communicated to all its details. The complicated system of an animal, which, when viewed without relation to its physiology, presented nothing but intricacy and confusion, will appear, when studied with reference to the purposes of its formation, as an elaborate machine, in which order and design are every where conspicuous, and which, in the assemblage and disposal of organs, and even in the construction of the minutest fibre, displays an exquisite and transcendent skill.

"As we had observed a gradation among the physical powers which actuate the different parts of the animal machine, so we may in like manner trace a regular subordination of the functions which they exercise. The great ends to which all the arrangements of the system, and all the movements of its parts evidently point, are the welfare and preservation of the individual being which they compose, and of the race to which it belongs. Sensation and voluntary motion are thus the primary objects to which all other functions are more or less directly subservient." (P. 33.)

The Lecture is of a nature too general and elementary for analysis; and, in recommending it to our readers, we are satisfied that they will derive both pleasure and instruction from its perusal.

---

